# Identification and immunological characterization of lipid metabolism-related molecular clusters in nonalcoholic fatty liver disease

**DOI:** 10.1186/s12944-023-01878-0

**Published:** 2023-08-09

**Authors:** Jifeng Liu, Yiming Li, Jingyuan Ma, Xing Wan, Mingjian Zhao, Yunshu Zhang, Dong Shang

**Affiliations:** 1 Department of General Surgery, The First Affiliated Hospital of Dalian Medical University, Dalian, Liaoning China; 2 Laboratory of Integrative Medicine, The First Affiliated Hospital of Dalian Medical University, Dalian, Liaoning China; 3 NHC Key Laboratory of Antibiotic Bioengineering, Laboratory of Oncology, Institute of Medicinal Biotechnology, Peking Union Medical College and Chinese Academy of Medical Sciences, Beijing, China; 4 Department of Plastic Surgery, The First Affiliated Hospital of Dalian Medical University, Dalian, Liaoning China

**Keywords:** Nonalcoholic fatty liver disease, Lipid metabolism, Immune infiltration, Machine learning, Biomarkers, Molecular clusters

## Abstract

**Background:**

Nonalcoholic fatty liver disease (NAFLD) is now the major contributor to chronic liver disease. Disorders of lipid metabolism are a major element in the emergence of NAFLD. This research intended to explore lipid metabolism-related clusters in NAFLD and establish a prediction biomarker.

**Methods:**

The expression mode of lipid metabolism-related genes (LMRGs) and immune characteristics in NAFLD were examined. The “ConsensusClusterPlus” package was utilized to investigate the lipid metabolism-related subgroup. The WGCNA was utilized to determine hub genes and perform functional enrichment analysis. After that, a model was constructed by machine learning techniques. To validate the predictive effectiveness, receiver operating characteristic curves, nomograms, decision curve analysis (DCA), and test sets were used. Lastly, gene set variation analysis (GSVA) was utilized to investigate the biological role of biomarkers in NAFLD.

**Results:**

Dysregulated LMRGs and immunological responses were identified between NAFLD and normal samples. Two LMRG-related clusters were identified in NAFLD. Immune infiltration analysis revealed that C2 had much more immune infiltration. GSVA also showed that these two subtypes have distinctly different biological features. Thirty cluster-specific genes were identified by two WGCNAs. Functional enrichment analysis indicated that cluster-specific genes are primarily engaged in adipogenesis, signalling by interleukins, and the JAK-STAT signalling pathway. Comparing several models, the random forest model exhibited good discrimination performance. Importantly, the final five-gene random forest model showed excellent predictive power in two test sets. In addition, the nomogram and DCA confirmed the precision of the model for NAFLD prediction. GSVA revealed that model genes were down-regulated in several immune and inflammatory-related routes. This suggests that these genes may inhibit the progression of NAFLD by inhibiting these pathways.

**Conclusions:**

This research thoroughly emphasized the complex relationship between LMRGs and NAFLD and established a five-gene biomarker to evaluate the risk of the lipid metabolism phenotype and the pathologic results of NAFLD.

**Supplementary Information:**

The online version contains supplementary material available at 10.1186/s12944-023-01878-0.

## Introduction

Nonalcoholic fatty liver disease (NAFLD) is now the major contributor to chronic liver disease, and the current global prevalence is 24% at present [1]. The liver pathology in NAFLD ranges from simple steatosis to nonalcoholic steatohepatitis (NASH) and can develop into fibrosis, cirrhosis, and hepatocellular carcinoma (HCC) [2]. NAFLD is becoming the fastest-expanding etiology of HCC [3]. Additionally, NAFLD individuals may also be at elevated risk of extrahepatic cancers, especially bladder cancer [4]. With the increasing prevalence of NAFLD, there has been a corresponding increase in clinical focus on its categorization. A liver biopsy is the most precise way to diagnose and subtype NAFLD, but technical problems or an unclear evaluation can reduce its effectiveness. Molecular subtype is a good addition to conventional histologic classification [5], and a complete molecular subtype assessment might be utilized in clinical evaluations. Thus, further accurate assessment of the NAFLD molecular subtype and the development of a predictive biomarker would be of significant clinical value.

Lipids play important roles in biological processes through their involvement in energy storage and metabolism and as signalling molecules for many cellular activities [6]. Lipids are involved in the course of numerous diseases, including but not limited to cardiovascular disease, obesity, diabetes, and cancer [7]. Various lipid changes caused by lipid metabolism disorders can lead to organelle dysfunction, such as lysosomal dysfunction, JNK activation, mitochondrial dysfunction, and ER stress, and eventually lead to cell death [8]. The systemic metabolism of lipids highly involves the liver. Lipid metabolism is closely related to nonalcoholic liver disease, and lipid metabolism disorder has a critical function in the progression of metabolic liver disease into nonalcoholic liver disease. Disruptions in hepatocyte lipid homeostasis lead to the production of toxic lipids, leading to dysfunctional organelles and promoting inflammation, hepatocyte damage, fibrosis and cell death [9]. Research has found that a few lipid moieties can mediate liver toxicity while facilitating hepatic inflammation, including leukotrienes, ceramides, fatty acids, and prostaglandins [10]. Excessive lipid uptake mediated by the lipid uptake-related factors FATP and CD36 promotes hepatic steatosis in NAFLD patients. Increased palmitate production during lipid production can lead to steatohepatitis through increased inflammation and apoptosis. However, the oxidation of fatty acids occurs mainly in mitochondria, and during this progress, a large amount of ROS is produced. ROS promote inflammation and nonalcoholic steatohepatitis progression. Elevated fatty acid levels promote ER stress, which inhibits the secretion of apolipoprotein B100, hinders the lipid transport process and promotes steatosis [11]. Moreover, studies have found that affecting the expression of lipid metabolism-related genes (LMRGs) can promote the progression of NAFLD [12]. Consequently, it is necessary to explore the LMRG function in NAFLD.

In recent years, RNA sequencing data analysis has become a functional tool for analysing gene expression. There have been several NAFLD-related bioinformatics studies [13, 14]. However, these studies usually only probe for differentially expressed genes (DEGs) and enrichment analyses. In contrast, this study performed a novel and thorough bioinformatics analysis, innovatively introduced LMRGs, further screened model candidate genes through two WGCNAs, and constructed a well-performing model by comparing multiple machine learning approaches. Specifically, the differentially expressed LMRGs (DE-LMRGs) between normal and NAFLD samples were first explored. Then, 71 NAFLD samples were divided into two LMRG-associated subgroups with significant biological functional differences. Next, the WGCNA algorithm was used to determine NAFLD-specific genes and cluster-specific genes and to investigate the biological roles and routes enriched by the intersecting genes. In addition, several machine learning algorithms were compared to construct a risk model. To verify the efficacy of the risk model, receiver operating characteristic (ROC) curves, nomograms, decision curve analysis (DCA), and test datasets were applied. Lastly, the potential mechanisms of biomarkers were analysed by gene set variation analysis (GSVA), and their relationship with immune cells was explored, thereby shedding new light on the prediction of NAFLD clusters and risk.

## Methods

### Data preparation

As of December 2022, the Gene Expression Omnibus (GEO) was searched for the keywords “NAFLD” and “nonalcoholic fatty liver disease”. After taking into account the sample size of the dataset and previous publications, four datasets (GSE48452, GSE89632, GSE126848, and GSE63067) were selected for this study [15–18]. Then, the GSE48452 and GSE89632 datasets, including 65 normal individuals and 71 NAFLD individuals, were selected as the training set for further analysis. Supplementary Table 1 concludes the clinical features of the 71 NAFLD individuals. The batch effects were addressed by employing the ComBat technique from the “SVA” package [19]. Moreover, the GSE126848 set (including 26 normal and 31 NAFLD individuals) and GSE63067 dataset (including 7 normal individuals and 11 NAFLD individuals) were selected as the test1 and test2 cohorts. In addition, 992 LMRGs (relevance score > 10) were obtained from the Gene Card Database. The LMRGs were further filtered by the DEGs. With adjusted *P* < 0.05 and FC > 1.5 as the criterion, DEGs were found by the “limma” program [20].

### Assessing immune cell infiltration

To compare the difference in immunity status between groups, ssGSEA from the “GSVA” package was applied to assess the proportions of several immune cell types [21]. The enrichment fraction of 28 immune cells was estimated for each individual according to the gene expression profile [22]. Then, the association between DE-LMRGs and immune cells was visualized. *P* < 0.05 indicated a significant link. Visualize using the “corrplot” R tool.

### Unsupervised clustering of NAFLD samples

Unsupervised clustering analysis of NAFLD patients was conducted using the “ConsensusClusterPlus” package based on the DE-LMRGs [23]. The k-means method with 1000 iterations was used to categorize 71 NAFLD individuals, and k was set to 9 to evaluate the suitable number of clusters.

### Gene module screening and coexpression network development

Utilizing the R package “WGCNA,“ WGCNA was carried out to find coexpression modules [24]. Using the best soft power, the weighted neighbor matrices were established and converted to a topological overlap matrix (TOM) [25]. The TOM dissimilarity metric was employed to construct modules when the minimum module size was adjusted to 100. In addition, genes with gene significance (GS) > 0.4 and module membership (MM) > 0.6 were considered specific genes.

### Analysis of functional enrichment

Functional enrichment studies were performed by Metascape, which was designed to provide an extensive resource for annotating and analyzing gene lists to investigate the biological roles and routes implicated in certain genes [26].

### Construction of machine learning models

Several models were built by the “caret” program, including the random forest model (RF), support vector machine model (SVM), eXtreme Gradient Boosting (XGB), and generalized linear model (GLM) [27–30]. By random selection, the 71 NAFLD samples were divided into a training set (70%) and a test set (30%). The characteristic significance and residual distributions of four models were identified by the “DALEX” package. The ROC curve was established using the “pROC” package [31]. After determining the best model, the 5 most important genes were considered the main predictive genes related to NAFLD. In addition, GSE126848 and GSE63067 were utilized to test the reliability of the biomarker.

### Establishment of a nomogram

The model genes were utilized for building a nomogram prediction model by “rms” R package [32]. All of the factors have a score associated with them, and the overall score is the aggregate of all predicted values. To calculate the prediction ability of the nomogram, the DCA was performed.

### Analysis of the model genes

GSVA was performed for model genes by the “GSVA” package [33]. It was recognized significantly altered if the |t| value was more than 2. The association between model genes and immunochemicals was evaluated on the basis of ssGSEA results.

### Single-cell data analysis

Two NAFLD samples (GSM4041162 and GSM4041163) were downloaded from the GSE136103 dataset [34]. The cells with more than 5% of mitochondrial genes or less than 50 genes expressed were removed, and genes expressed in at least three cells were selected [35]. After data preprocessing, the “NormalizeData” function in R was used to normalize the data. The “SingleR” program was used to note cell types [36].

## Results

### Dysregulation of LMRGs and immune responses in NAFLD

**Fig. 1:**
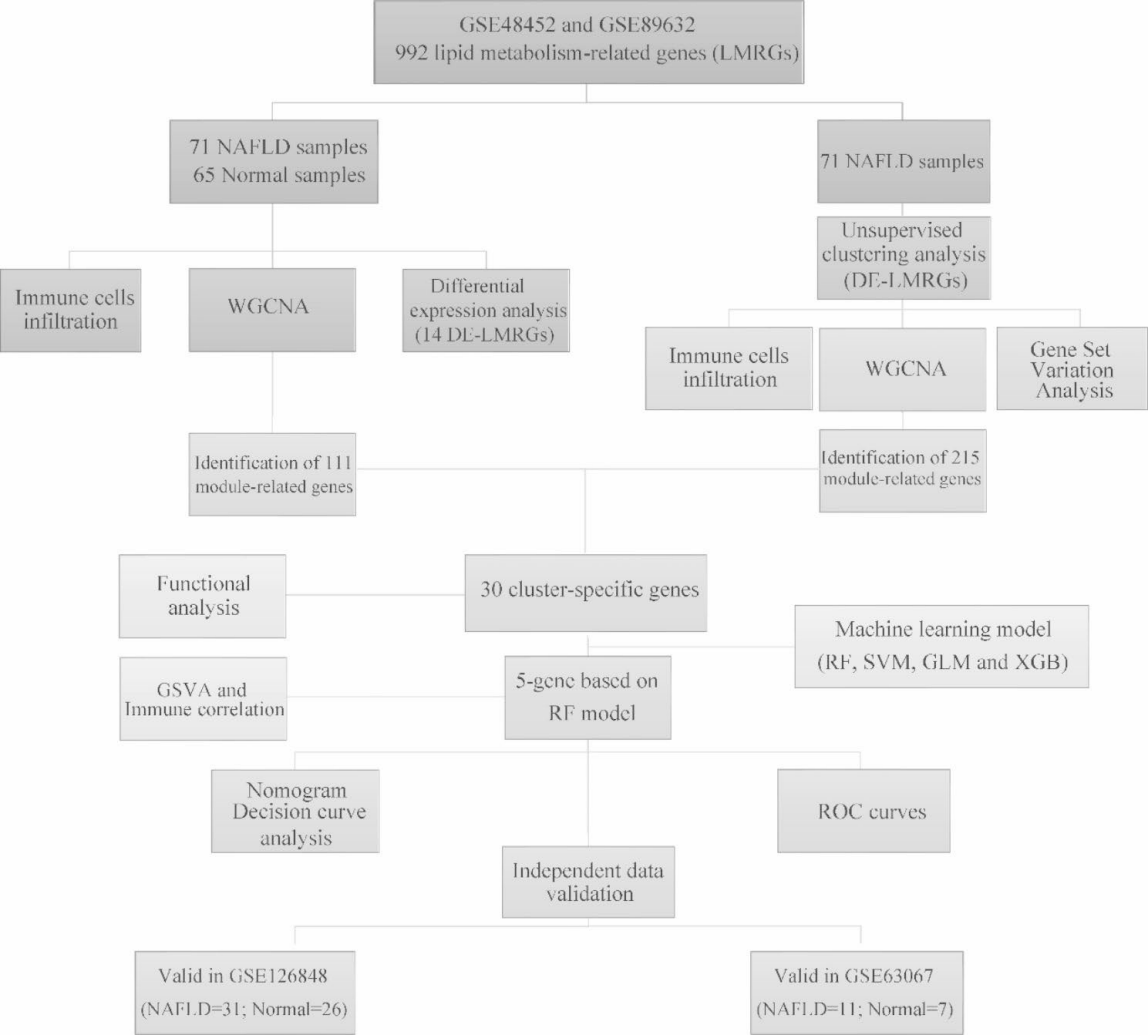
The analytical workflow of the research in detail

**Fig. 2:**
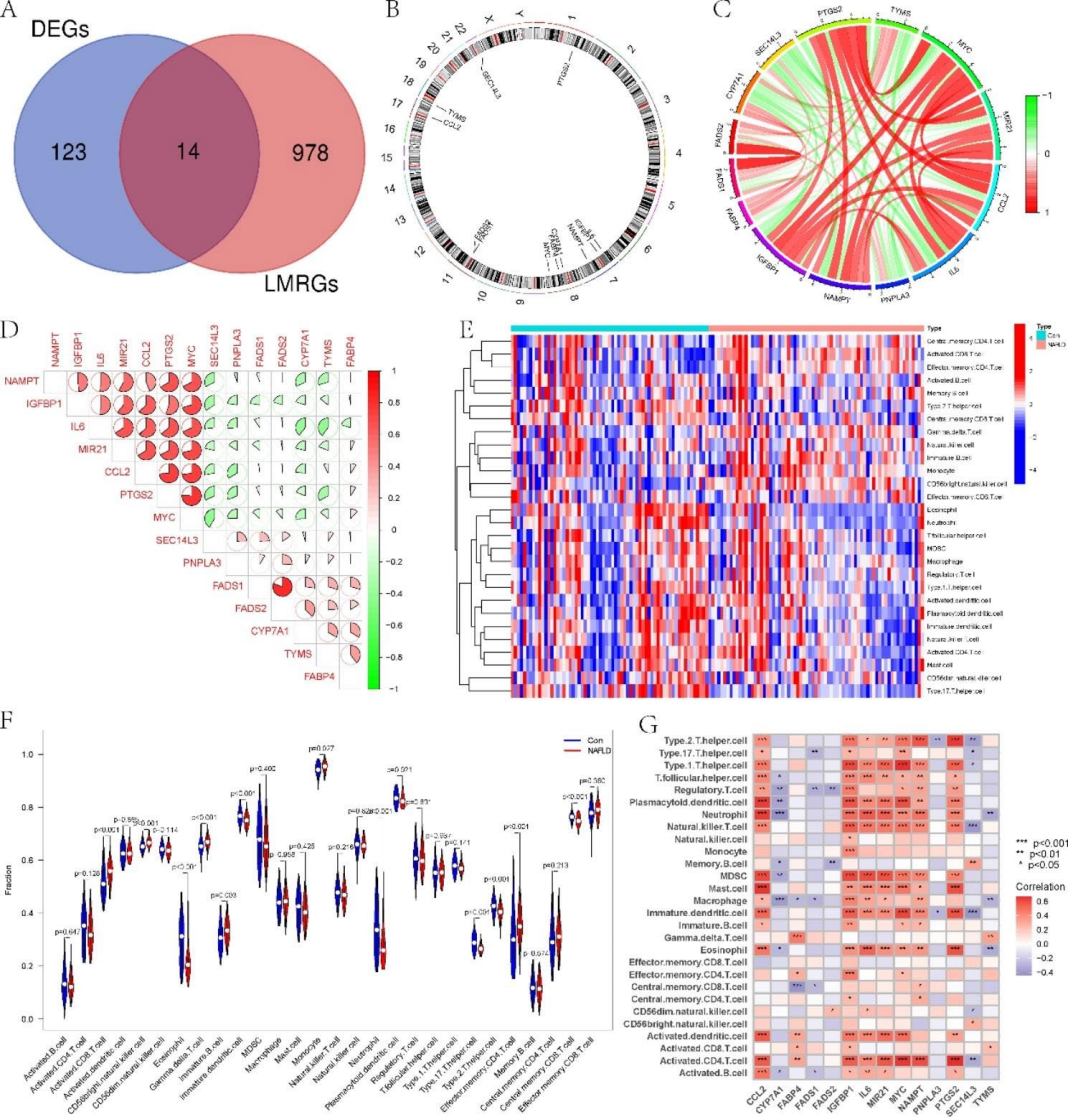
Fourteen DE-LMRGs in NAFLD. (**A**) Intersection plot of the LMRGs and the DEGs. (**B**) Location of the LMRGs in chromosomes. (**C-D**) Network diagram of the 14 LMRGs. (**E**) Heatmap of the immune infiltration of NAFLD and normal individuals analysed using ssGSEA. (**F**) Comparison of immune infiltration between NAFLD and normal individuals. (**G**) Relationship between LMRGs and immune cells

Figure 1 illustrates the flow chart for the work. To elucidate the biological roles of lipid metabolism in NAFLD, the expression of 992 LMRGs was thoroughly compared between NAFLD and normal samples. Fourteen LMRGs in total were shown to have differential expression (Fig. 2A). Figure 2B displays the location of LMRGs in chromosomes. Then, a correlation analysis was performed between 14 DE-LMRGs. The gene relationship network diagram showed that there were associations between different LMRGs (Fig. 2C-D).

In addition, NAFLD patients presented higher infiltration levels of effector memory CD4 T cells, CD56 bright NK cells, gamma delta T cells, immature B cells, monocytes, and activated CD8 T cells. The number of eosinophils, immature dendritic cells, neutrophils, plasmacytoid dendritic cells, T helper cells, and central memory CD8 T cells were higher in normal individuals (Fig. 2E, F). Meanwhile, many immune cells, mainly T helper cells, NK T cells, and activated CD4 T cells, were found to be closely related to LMRGs (Fig. 2G).

### LMRG-related subgroups in NAFLD

**Fig. 3:**
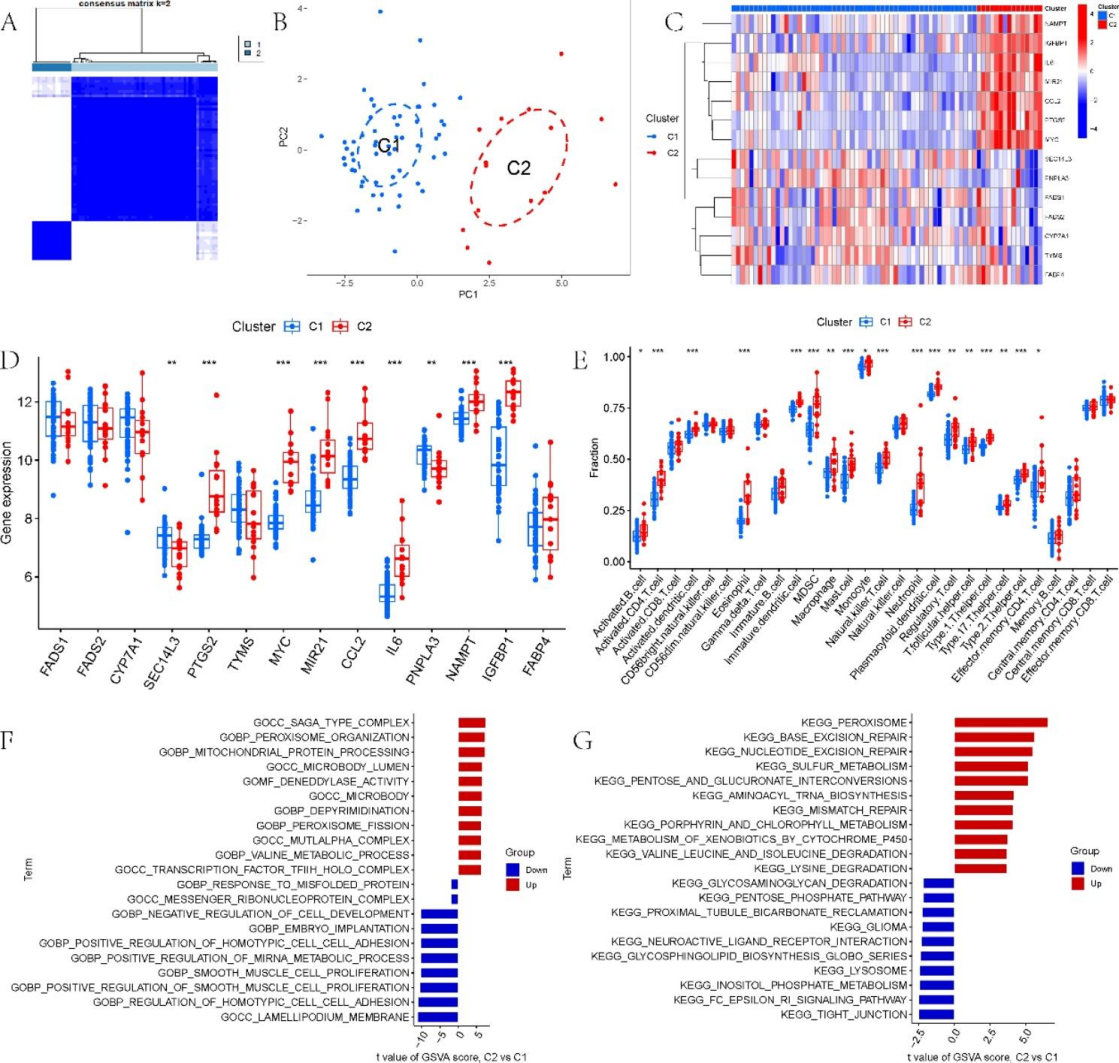
LMRG-related subgroups in NAFLD. (**A**) Consensus clustering matrix with k = 2. (**B**) PCA showing the subtype distribution. (**C**) The expression of fourteen LMRGs in C1 and C2. (**D**) The expression of fourteen LMRGs in C1 and C2. (**E**) Comparison of immune cell infiltration between C1 and C2. (**F-G**) GSVA of two LMRG clusters

71 NAFLD samples were categorized into two subgroups on the basis of 14 DE-LMRG expression profiles (Fig. 3A). Principal component analysis (PCA) showed significant differences between C1 and C2 (Fig. 3B). C1 revealed high expression levels of SEC14L3 and PNPLA3, while PTGS2, MYC, MIR21, CCL2, IL6, NAMPT, and IGFBP1 were more highly expressed in C2 (Fig. 3C, D). It was also found that C2 had noticeably more immune cell infiltration than C1 (Fig. 3E). In addition, the GSVA showed that C2 was mainly enriched in SAGA type complex, peroxisome organization, deneddylase activity, peroxisome, base excision repair, and nucleotide excision repair (Fig. 3F, G).

### Gene module screening and coexpression network development

**Fig. 4:**
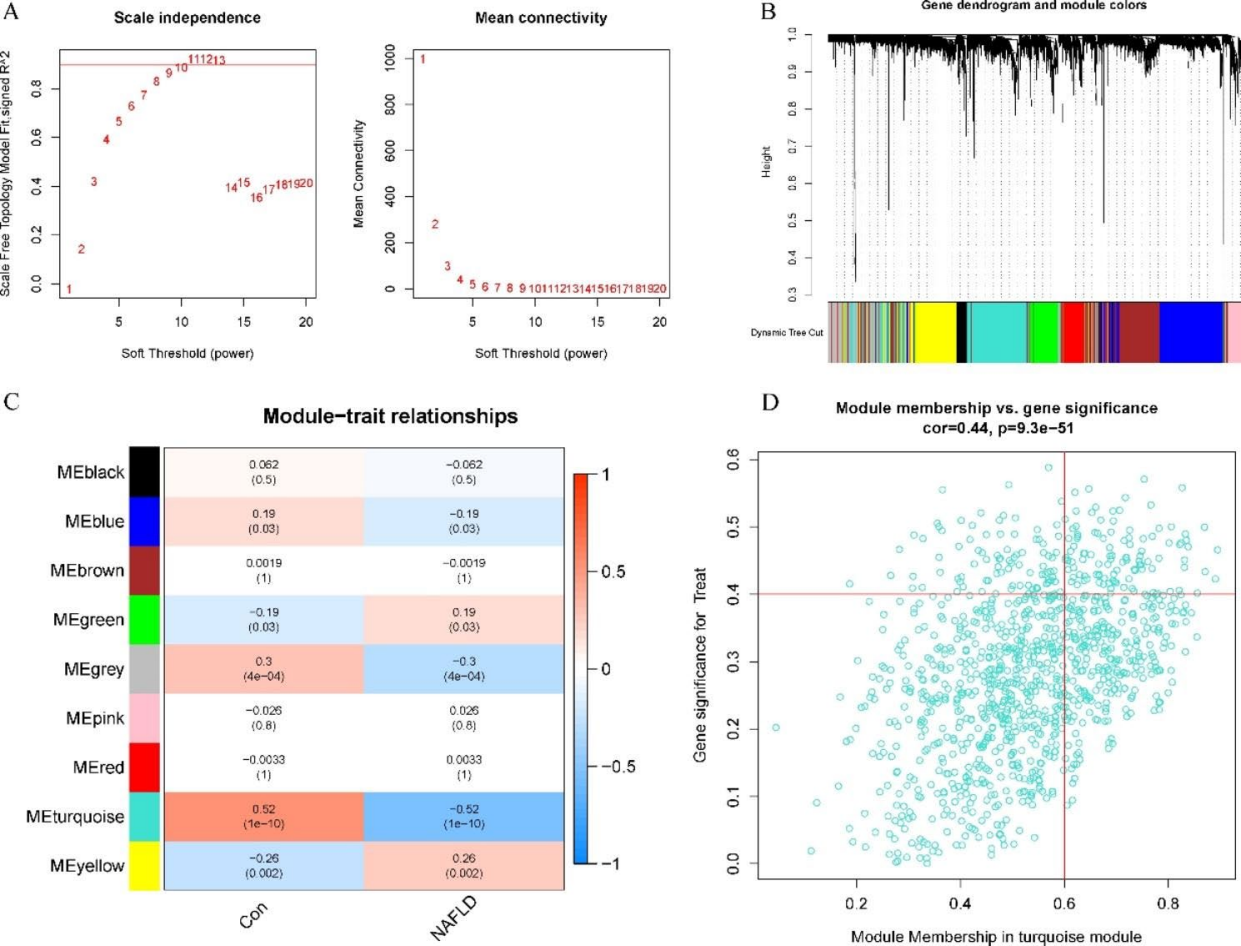
Coexpressed network in NAFLD-normal samples. (**A**) Soft-threshold power selection. (**B**) Correlation heatmap for nine modules. (**C**) Eigengene correlation with clinical status. (**D**) Scatter plot of turquoise module genes

WGCNA was utilized to discover the important modules related to NAFLD. The scale-free R^2^ parameter was set to 0.9, and the soft power parameter was set to 9 to identify coexpressed gene modules (Fig. 4A). The method of dynamic cutting was utilized to acquire nine different coexpression modules (Fig. 4B). The turquoise module displayed the highest relevance (Fig. 4C). The hub genes in the turquoise module were chosen for subsequent analysis (Fig. 4D).

**Fig. 5:**
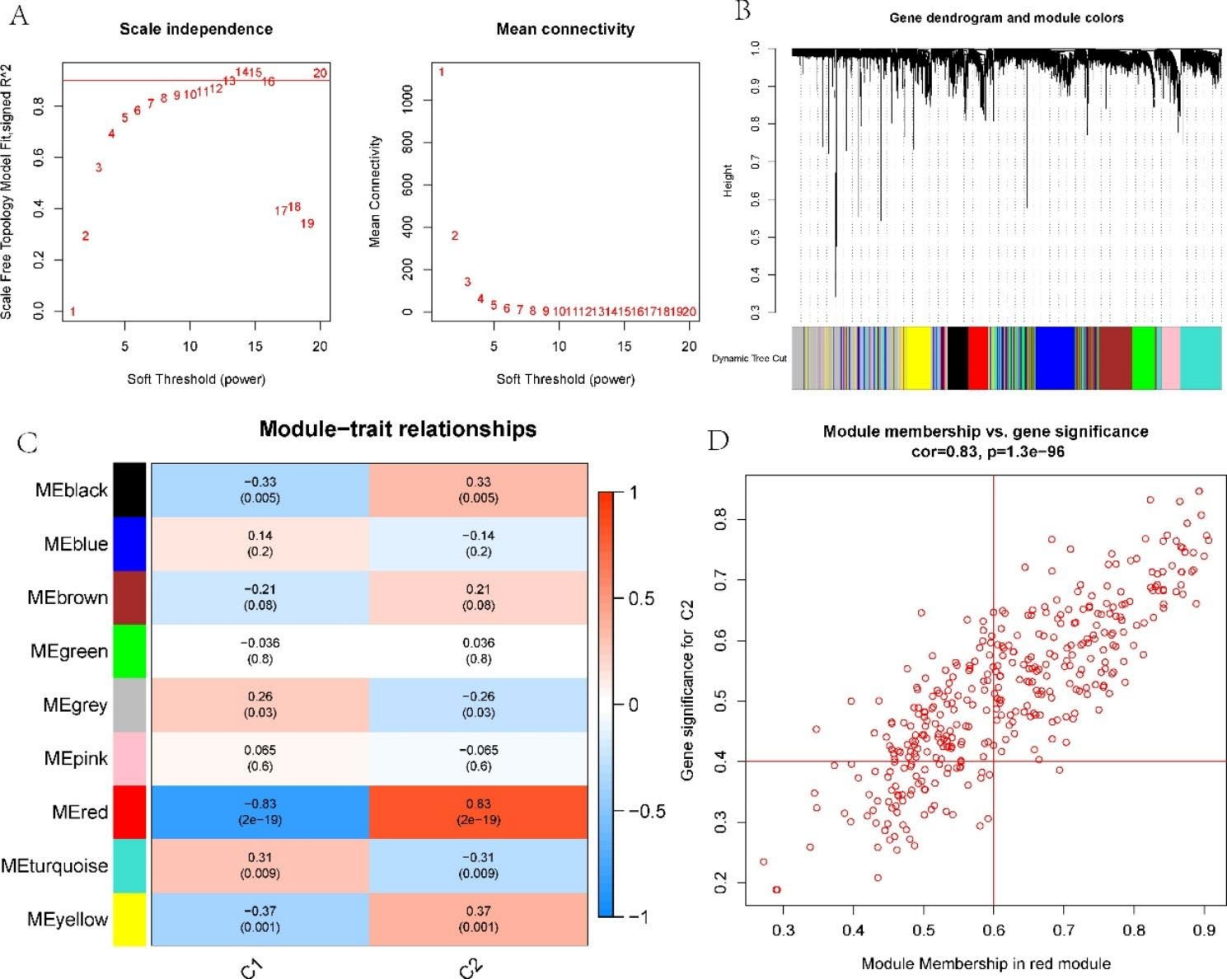
Coexpression network of LMRG-related clusters. (**A**) Soft-threshold power selection. (**B**) Correlation heatmap for nine modules. (**C**) Correlation analysis of clinical state and module eigengenes. (**D**) Scatter plot of red module genes

Furthermore, WGCNA was also utilized to evaluate the essential modules that were highly connected with LMRG-related clusters (Fig. 5A). The method of dynamic cutting was utilized to acquire nine different coexpression modules (Fig. 5B). The red module had the highest connection with LMRG-related clusters (Fig. 5C). Similarly, the red module’s pivotal genes were chosen for subsequent analysis (Fig. 5D).

### Functional enrichment analysis

**Fig. 6:**
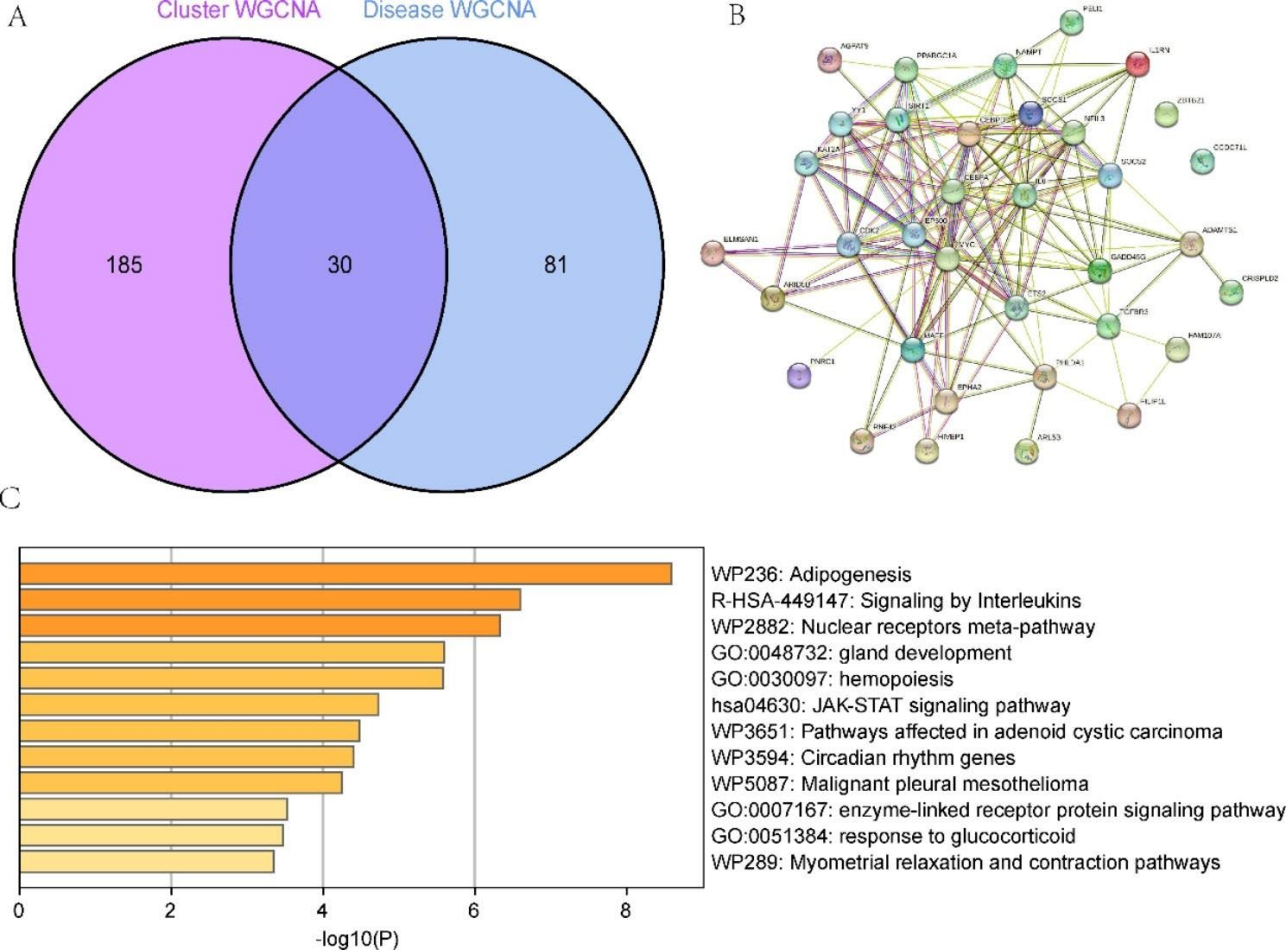
Functional enrichment analysis. (**A**) Intersection of hub genes. (**B**) PPI network of the 30 cluster-specific genes. (**C**) Functional enrichment analysis of cluster-specific genes

Intersection of the module-associated genes of LMRG-related clusters with the module-associated genes of NAFLD and normal samples was performed, and a total of 30 cluster-specific genes were found (Fig. 6A). PPI analysis showed that except for CCDC71L and ZBTB21, the other 28 genes were closely intertwined (Fig. 6B). In addition, the Metascape results showed marked enrichment in adipogenesis, signalling by interleukins, and the nuclear receptor meta-pathway (Fig. 6C).

### Development of machine learning models

**Fig. 7:**
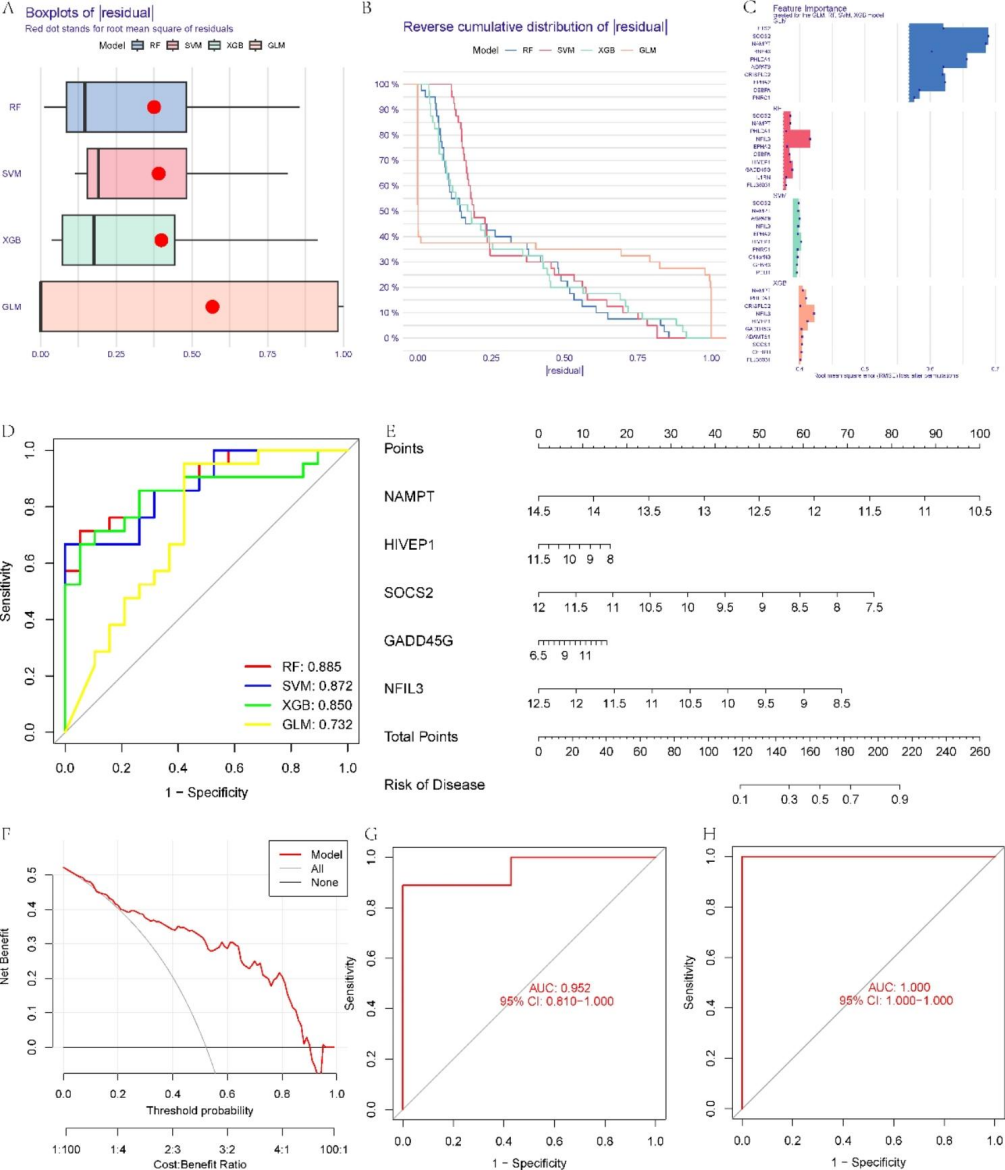
Four different machine learning models. (**A**) Boxplots display the residuals of each model. The red dot represents the root mean square of the residuals. (**B**) Distribution of cumulative residuals for different models. (**C**) The crucial components of each model. (**D**) ROC curve of the 4 models in the training cohort. (**E**) Nomogram for forecasting NAFLD. (**F**) DCA plot to measure the prediction capability of the model. (**G, H**) ROC curve in the GSE126848 (**G**) and GSE63067 (**H**) datasets

According to the 30 cluster-specific genes, four validated machine learning models were created. The “DALEX” program was used to describe the four models and depict the residual distribution of each model. The RF machine learning model had comparatively less residual variance (Fig. 7A, B). The 10 most crucial genes for each model were then rated (Fig. 7C). Additionally, ROC curves were calculated to determine the prediction ability of the four models, and the RF model demonstrated the best performance (Fig. 7D). In conclusion, these findings suggest that the RF machine learning model was most effective in differentiating NAFLD with distinct clusters. Finally, the top five most significant variables of the RF model (NAMPT, HIVEP1, SOCS2, GADD45G, and NFIL3) were selected as predictor model genes for subsequent analysis.

### Construction of the nomogram

For further evaluating the risk of NAFLD individuals, a nomogram prediction model was developed utilizing model genes (Fig. 7E). Then, the prediction capacity of the nomogram was evaluated by DCA. DCA suggested that the nomogram had excellent accuracy, which may aid clinical decision-making (Fig. 7F). Then, GSE126848 and GSE63067 were used to verify the prediction model. The five-gene biomarker exhibited promising performance with an AUC value of 0.952 in GSE126848 and 1.000 in GSE63067 (Fig. 7G, H).

### Analysis of five model genes

GSVA was applied to study the biological function of five model genes in NAFLD. GADD45G was mainly upregulated in maturity-onset diabetes of the young and metabolism and downregulated in the B-cell receptor (BCR), NOD-like receptor (NLR), and Toll-like receptor (TLR) signalling routes (Supplementary Fig. 1A). HIVEP1 was mainly upregulated in mismatch repair and DNA replication and downregulated in the cytokine‒cytokine receptor interaction (CCRI), TLR, and NLR signalling pathways (Supplementary Fig. 1B). NAMPT was mainly upregulated in sulfur metabolism and base excision repair and downregulated in the CCRI, TLR, and BCR signalling pathways (Supplementary Fig. 1C). NFIL3 was mainly upregulated in mismatch repair and sulfur metabolism and downregulated in the CCRI, haematopoietic cell lineage, and BCR signalling pathways (Supplementary Fig. 1D). SOCS2 was mainly upregulated in mismatch repair, sulfur metabolism and DNA replication and downregulated in primary immunodeficiency, NLR, and BCR signalling pathways (Supplementary Fig. 1E). Furthermore, given that cluster-specific genes were negatively associated with NAFLD, these five model genes may inhibit the progression of NAFLD primarily by suppressing multiple inflammatory and immune-related pathways.

**Fig. 8:**
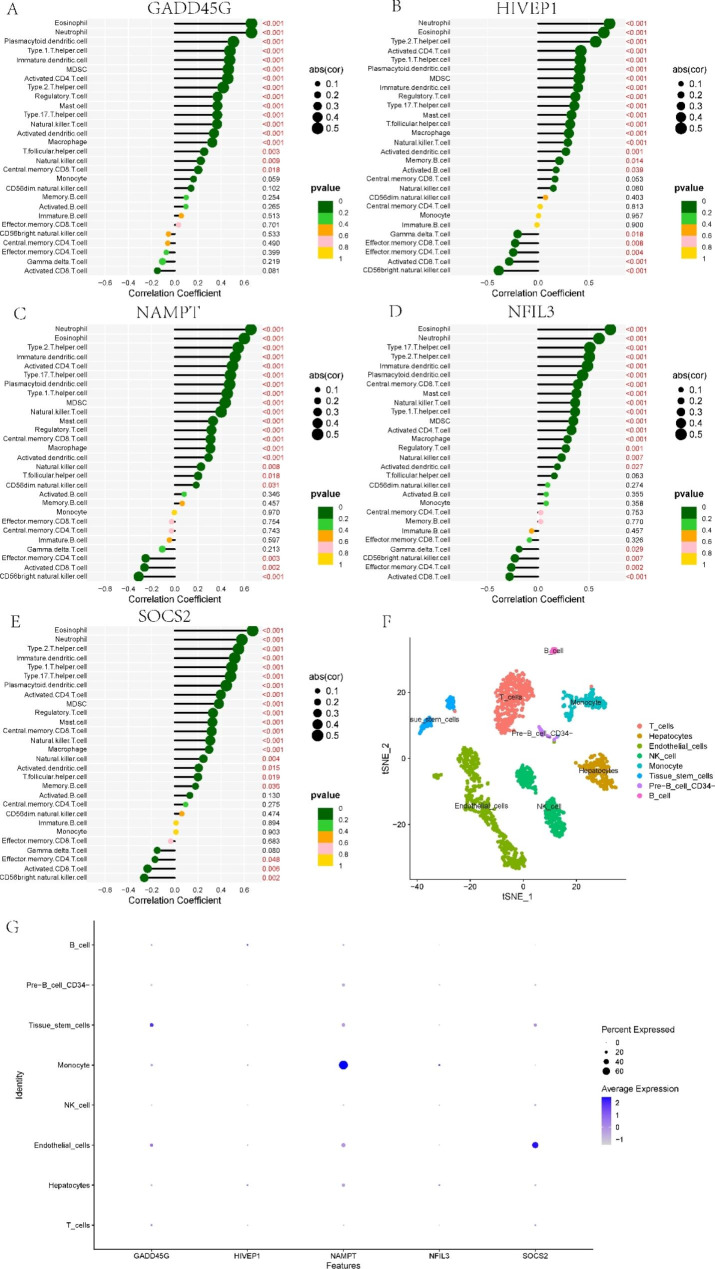
Correlations between infiltrating immune cells and GADD45G (**A**), HIVEP1 (**B**), NAMPT (**C**), NFIL3 (**D**), and SOCS2 (**E**). (**F**) Annotation of different cell types. (**G**) Expression levels of five genes in different cell types

Then, the relationship between five model genes and immunochemicals was explored. The results revealed that they were mainly positively related to eosinophils, neutrophils, and T helper cells and negatively correlated with CD56-bright NK cells, effector CD4 T cells, and activated CD8 T cells (Fig. 8A-E). To investigate the expression of model genes in specific cell populations, publicly available scRNA-seq data from two NAFLD individuals were utilized for analysis. They were clustered and annotated into eight different cell types (Fig. 8F). GADD45G was mainly expressed in tissue stem cells, NAMPT was mainly expressed in monocytes, and SOCS2 was mainly expressed in endothelial cells (Fig. 8G).

## Discussion

NAFLD is the major contributor to chronic liver disease around the world. A model of epidemiology predicted that the prevalence of NAFLD would keep rising and the death rate from linked diseases would double by 2030 [37]. The pathogenesis of NAFLD is complex and is thought to rely on multiple parallel hits in the context of genetic susceptibility [38]. Lipids are a class of structurally diverse hydrophobic molecules with multiple functions. For example, lipids are essential energy storage molecules that contribute to the formation of cell membranes, participate in many signal transduction cascade reactions and have a caloric output of 9 kcal/g compared to the 4 kcal/g for proteins and carbohydrates [10]. Lipid metabolism has a critical effect on NAFLD, especially in metabolic liver disease [9]. Lipid metabolism-related factors can participate in regulating lipid metabolism in a variety of ways [11]. Additionally, lipid metabolism disorders can lead to dysfunctional organelles and promote inflammation, liver cell damage and cell death and a series of consequences. An essential component of NAFLD is lipotoxicity. Lipotoxicity of hepatocytes is also proportional to the severity of NAFLD. In NAFLD, hepatocytes accumulate triglycerides and different lipid byproducts, like free cholesterol, ceramides, and free fatty acids (FFAs). FFAs are considered the main mediator of hepatocyte lipotoxicity [10]. Moreover, it has been reported that changes in LMRG expression can regulate lipid metabolism and participate in the progression of NAFLD [13]. Therefore, the work intended to explore the precise effect of LMRG on the NAFLD phenotype and immune microenvironment. Additionally, LMRGs were used to predict NAFLD subtypes and construct reliable disease prediction models.

To illustrate the essential function of LMRGs in NAFLD, a thorough analysis of the expression profiles of LMRGs was first performed between normal and NAFLD individuals. Fourteen LMRGs were discovered to differentially express, indicating that these LMRGs have an essential role in NAFLD. To further comprehend the correlation between LMRGs and NAFLD, the correlations among LMRGs were calculated. The results showed that most LMRGs have synergistic or antagonistic effects. It is widely known that immune cells have diverse but essential roles in the inflammatory processes of NAFLD [39]. Therefore, NAFLD and normal tissue immune cell infiltration levels were contrasted. NAFLD patients had greater levels of CD8 T cell, monocyte, NK cell, B cell, and CD4 T-cell infiltration. According to reports, these immune cells are strongly related to the development of NAFLD. For example, infiltration of B cells is involved in chronic liver diseases [40]. Moreover, CD8 T cells may participate in the progression and regression of liver fibrosis [41].

Next, two independent subgroups with significant biological functional differences were discovered to accentuate the diverse patterns of lipid metabolism in NAFLD individuals. C2 had noticeably more immune cell infiltration than C1, suggesting that C2 may have more activated immune cells to prevent the development of NAFLD and thus have a better prognosis. A promising approach for assessing prognosis and managing individuals with NAFLD involves risk stratification based on LMRG.

While molecular typing is essential for functional mining of LMRGs, it has some shortcomings in type clustering that make it difficult to accurately predict clinical outcomes and risk scores for individual patients. To address this issue, WGCNA and machine learning were used to create a prediction model comprising five genes that had excellent performance in predicting NAFLD. More importantly, the five-gene RF model was able to accurately predict NAFLD in two test sets, providing new insights for the early diagnosis of NAFLD. Additionally, a nomogram was created for the diagnosis of NAFLD using SOCS2, NAMPT, GADD45G, HIVEP1, and NFIL3. The model demonstrated strong predictive value, as evidenced by the ROC and DCA curves.

Other researchers have also studied the 5 genes and discovered that they have a crucial role in NAFLD and other diseases. SOCS2 is an inflammatory modulator. It can control obesity by regulating adipose tissue [42]. Moreover, SOCS2 in macrophages inhibits inflammation and apoptosis by suppressing the NF-κB signalling route and plays a negative regulatory role in inflammation and apoptosis during NAFLD. Therefore, it can be used as a potential preventive and therapeutic target for NAFLD [43]. NAMPT is able to regulate the pathogenesis of obesity and related diseases, especially NAFLD, by affecting lipid and glucose metabolism, inflammation and apoptosis [44]. GADD45G is shown to be a novel tumour suppressor in acute myeloid leukaemia as a corresponding gene for DNA damage [45]. However, its role in NAFLD has not been clearly reported. HIVEP1 is a negative regulator of NF-κB, inhibiting the pro-inflammatory responses to bacterial agonists [46]. Therefore, it may have an ameliorating effect on inflammation in NAFLD. NFIL3 is an important transcriptional regulator of immune cell growth and differentiation as well as a key regulator of hepatic glucose homeostasis. Enhancing hepatic NFIL3 activity in insulin-resistant conditions is advantageous for reducing glycaemic symptoms in metabolic disorders [47]. In addition, NFIL3 is an important molecular link between the microbiota, biological clock and host metabolism. It was shown that the microbiota regulates lipid uptake and storage through NFIL3 [48]. Therefore, NFIL3 might be used as a treatment target for metabolic illnesses, including NAFLD.

In addition, GSVA was performed to explore the biological function of biomarkers in NAFLD. It was discovered that five model genes are mainly downregulated in some inflammatory and immune-related pathways, suggesting that they may inhibit NAFLD disease progression through these routes. Further immune cell correlation analysis identified that the model genes were strongly related to eosinophils and neutrophils. Collectively, lipid metabolism might promote NAFLD flare-ups and progression by influencing the inflammatory response and immune microenvironment.

### Study strengths and limitations

This is the first bioinformatics study to extensively examine the role of LMRGs in NAFLD. However, there have been several previous bioinformatics studies related to NAFLD [49, 50]. In this study, the LMRGs were innovatively used as the grouping basis, and the hub genes were further determined through two WGCNA analyses. In addition, four machine learning models were compared to obtain the key predicted genes, which further reduced the model error. However, this research has a few limitations that should be acknowledged. First, this study was retrospective and was performed using mainly data from public databases. Therefore, the prediction capability of the model should be validated in prospective clinical research with large samples. Second, further investigation of molecular mechanisms is required to explore the function of model genes and lipid metabolism in the occurrence and development of NAFLD.

## Conclusion

This study demonstrated the relationship between LMRGs and immune cell infiltration and significant immunological heterogeneity between NAFLD individuals with different lipid metabolism subgroups. In addition, a diagnostic model on the basis of LMRG was created, which will contribute to the early clinical diagnosis and management of NAFLD.

### Electronic supplementary material

Below is the link to the electronic supplementary material.

Additional file: Fig. 1. GSVA of GADD45G (A), HIVEP1 (B), NAMPT (C), NFIL3 (D), and SOCS2 (E).

Additional file: Table 1. The clinical features of 71 NAFLD individuals.

## Data Availability

The datasets analysed in this work may be found in the GEO databases. Additionally, any raw data and analytic technologies can be requested by directly contacting the author if the request is reasonable.
