# Supplementary material for: Identification and immunological characterization of lipid metabolism-related molecular clusters in nonalcoholic fatty liver disease
Source: Lipids Health Dis. 2023 Aug 9;22:124. doi: 10.1186/s12944-023-01878-0 (PMC10410946; doi:10.1186/s12944-023-01878-0)
Supplement: Supplementary file 2 — Additional file: Table 1. The clinical features of 71 NAFLD individuals. [file 12944_2023_1878_MOESM2_ESM.docx]

|  | GSE48452 (n=32) | GSE89632 (n=39) | Total (n=71) |
| --- | --- | --- | --- |
| Age (years) | 43.75±9.85 | 44.10±10.90 | 43.96±10.44 |
| Male Sex (% (n/n)) | 25.00 (8/32) | 58.97 (23/39) | 43.66 (31/71) |
| BMI (kg/m^2^) | 46.98±10.52  (BMI≥30, n=30) | 30.24±5.03  (n=37; BMI≥30, n=16) | 38.00±11.60  (n=69; BMI≥30, n=46) |
| Triglycerides (mmol/L) | - | 1.96±1.90  (n=34; TG≥1.7, n=16) | - |
| Visceral adiposity index (VAI) | - | 2.30±1.30 (n=29) | - |
| NAS score | 3.59±1.88 | 3.26±1.87 (n=38) | 3.41±1.87(n=70) |
